# Dataset of various characterizations for novel bio-based plastic poly(benzoxazole-co-benzimidazole) with ultra-low dielectric constant

**DOI:** 10.1016/j.dib.2019.104114

**Published:** 2019-06-08

**Authors:** Aniruddha Nag, Mohammad Asif Ali, Makoto Watanabe, Maninder Singh, Kittima Amornwachirabodee, Shunsuke Kato, Tetsu Mitsumata, Kenji Takada, Tatsuo Kaneko

**Affiliations:** a Graduate School of Advanced Science and Technology, Energy and Environment Area, Japan Advanced Institute of Science and Technology (JAIST), 1-1 Asahidai, Nomi, Ishikawa, 923-1292 Japan; b Department of Materials Science & Technology, Faculty of Engineering, Niigata University, Ikarashi, Nishi-ku, Niigata, 950-2181, Japan

**Keywords:** Characterization, High-performance, Polymer, Dielectric

## Abstract

The data presented in this specified data article comprise of various characterization such as: structural, thermal, elemental etc. to understand the novel structure and specific properties of the bio-based plastic as described in the main research article “High-performance poly (benzoxazole/benzimidazole)bio-based plastics with ultra-low dielectric constant from 3-amino-4-hydroxybenzoic acid” [1]. The data of ^1^H NMR spectra of two monomers and their HCl salt formation required for polymerization, FT-IR spectra of polymer formation before and after thermal ring-closing and additionally supported by the thermogravimetric plots where mass loss due to water is observed around 400 °C (thermal ring closing temperature). Solvent plays effective role to change dielectric properties significantly, complete removal of the remaining solvents was confirmed by X-ray photoelectron spectroscopy (XPS) technique. Wide-angle XRD dataset was presented here to make an idea about degree of crystallinity of the prepared polymers.

**Table:** Specifications table

Subject area	Chemistry
More specific subject area	Bio-based polymer
Type of data	NMR, FT-IR spectra, TGA plots, XPS data, XRD plots, sample image
How data was acquired	^1^H NMR was performed on a ‘BRUKER Biospin AG 400 MHz spectrometer’. FT-IR spectra were recorded with a ‘Perkin-Elmer Spectrum One spectrometer’ between 4000 and 400 cm^−1^ using a diamond-attenuated total reflection (ATR). Thermo-gravimetric analysis (TGA) was performed on a ‘HITACHI STA7200’. The samples (<5 mg) were placed in a platinum crucible heated to a maximum temperature of 800 °C at a heating rate of 10 °C min^−1^ under a nitrogen atmosphere. The XRD patterns of the samples were analyzed using X-ray diffraction (RIGAKU Smartlab) operated at 40 kV, 30 mA with Cu-Kα radiation (1.5418 Å). XPS were measured using ‘SHIMADZU KRATOS Axis-Ultra DLD’ instrument.
Data format	Raw and analyzed
Experimental factors	^1^H NMR was performed using DMSO-*d*_6_ as solvent at 23.1 °C. FT-IR spectra were recorded using powder. In case of TGA to observe thermal ring closing behavior we have pre-heated the sample which is designated as PBI-*co*-PBI, in case of pre-(PBO-*co*-PBI) no pre-heating was done. Polymer powder was used in both the cases of XPS and XRD analysis.
Experimental features	Various compositions of pre-PBO-*co*-PBI were obtained using 3-amino-4-hydroxybenzoic acid (3, 4-AHBA) and 3, 4-diaminobenzoic acid (3, 4-DABA) in different compositions. PBO-*co*-PBI was obtained by stepwise heating to get completely cyclized oxazole and imidazole rings.
Data source location	School of Materials Science, Japan Advanced Institute of Science and Tech-nology (JAIST), 1-1 Asahidai, Nomi-shi, Ishikawa, 923–1292, Japan 36°26′40.3″N, 136°35′33.5″E 36.444528, 136.592639
Data accessibility	Data is with this article.
Related research article	A. Nag, M. A. Ali, M. Watanabe, M. Singh, K. Amornwachirabodee, S. Kato, T. Mitsumata, K. Takada, T. Kaneko, High-performance poly (benzoxazole/benzimidazole)bio-based plastics with ultra-low dielectric constant from3-amino-4-hydroxybenzoic acid, *Polym. Degrad. Stab.* 162 (2019), 29–35 [1].

**Table:** 

**Value of the data** • The following ^1^H NMR data are useful to understand the difference between the proton peaks before and after salt formation, which is necessary for polycondensation of both monomer. • Understanding of thermal ring closing using via FT-IR spectra and observing weight-loss during thermal cyclisation which comprises water elimination. • Elemental analysis using XPS technique is useful to understand presence of trace amount of solvent which can affect the polymer properties in multiple folds. • XRD pattern of the copolymer PBO-*co*-PBI and homopolymers (PBO or PBI) can be useful to refer by other researchers.

## 1 Data

**Fig. 1:**
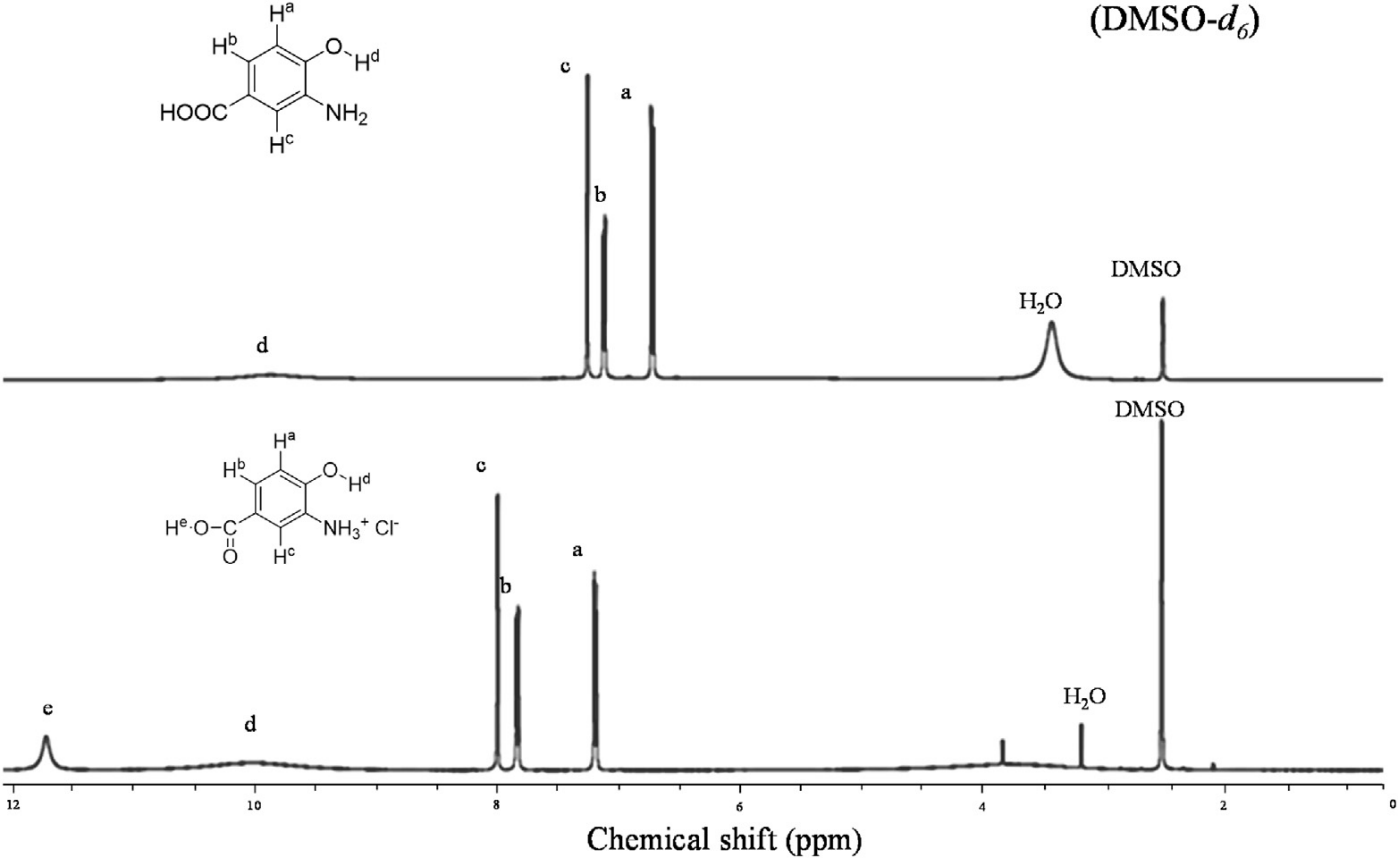
^1^H- NMR spectra of 3, 4-AHBA and 3, 4-AHBA.HCl.

In ^1^H NMR spectra (Fig. 1) comparison between monomer 3, 4-AHBA and its salt i.e. 3, 4-AHBA.HCl, the main chain proton signals for aromatic protons appeared between 6.7 and 7.3 ppm, while after salt preparation peak shift occurred and the corresponding proton signals were placed between 7.2 and 8.0 ppm.

On the other hand, aromatic hydroxyl (-OH) peak position remains same as broad singlet between 9.5 and 10.5 ppm. Aromatic carboxylic acid (-COOH) peak appeared after the salt preparation at 11.6–11.8 ppm.

**Fig. 2:**
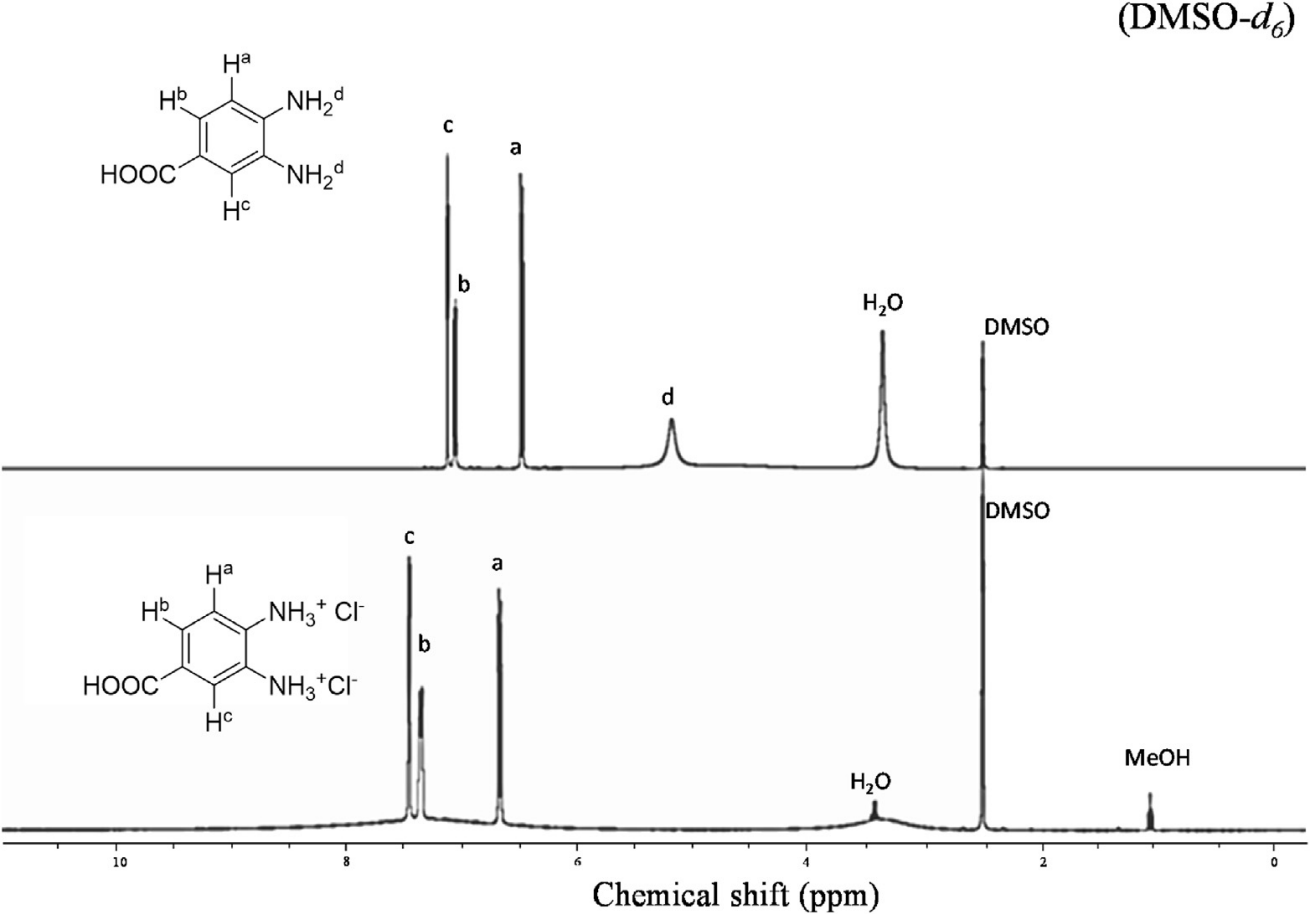
^1^H- NMR spectra of 3,4-DABA and 3,4-DABA.2HCl.

Fig. 2 shows the ^1^H NMR spectra comparison between monomer 3, 4-DABA and its salt 3, 4-DABA. 2HCl, where the aromatic proton signals appeared in between 6.4 and 7.2 ppm, while after salt preparation peaks shifted in between 6.7 and 7.5 ppm. More significantly diamine proton peaks clearly observed at 5.2 ppm and disappeared after salt formation, which confirms the diamine salt structure.

**Fig. 3:**
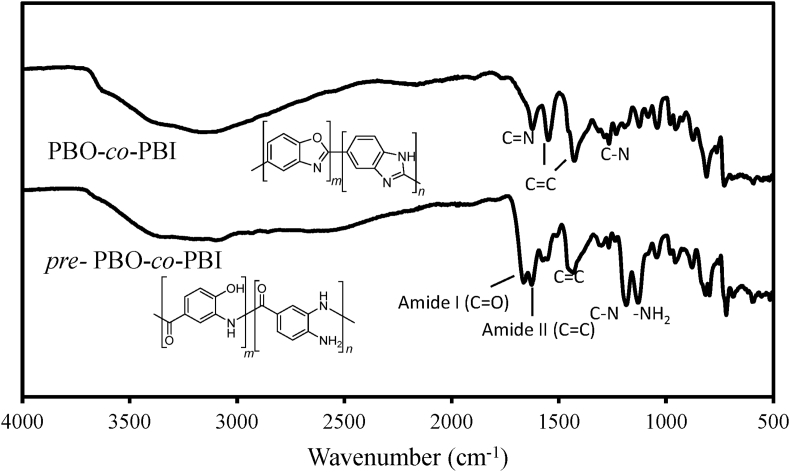
FT-IR spectra of PBO-*co*-PBI and its corresponding precursor *pre*-PBO-*co*-PBI.

Fig. 3 shows the FT-IR spectra comparison between *pre*-PBO-*co*-PBi and PBO-*co*-PBI.

The stretching vibration of *pre*- PBO-*co*-PBI spectra shows two peaks for the carbonyl (C=O) at 1673 cm^−1^ (amide I) and 1634 cm^−1^ (amide II) bands, which were disappeared at 400 °C indicating that PBO-*co*-PBI has no carbonyl (C=O) group available in the structure. However, the absorption band at 1458 cm^−1^ was directly attributed to the characteristic C=C indicates stretching of aromatic bands and other absorption bands observed at 1197 cm^−1^, and 1140 cm^−1^ were attributed to the characteristic C–N bonds and –NH_2_ group were disappeared after thermal cyclization. The distinct characteristic C=N and C–N absorption around 1624 cm^−1^ and 1292 cm^−1^ were associated to oxazole and imidazole ring [2], [4].

**Fig. 4:**
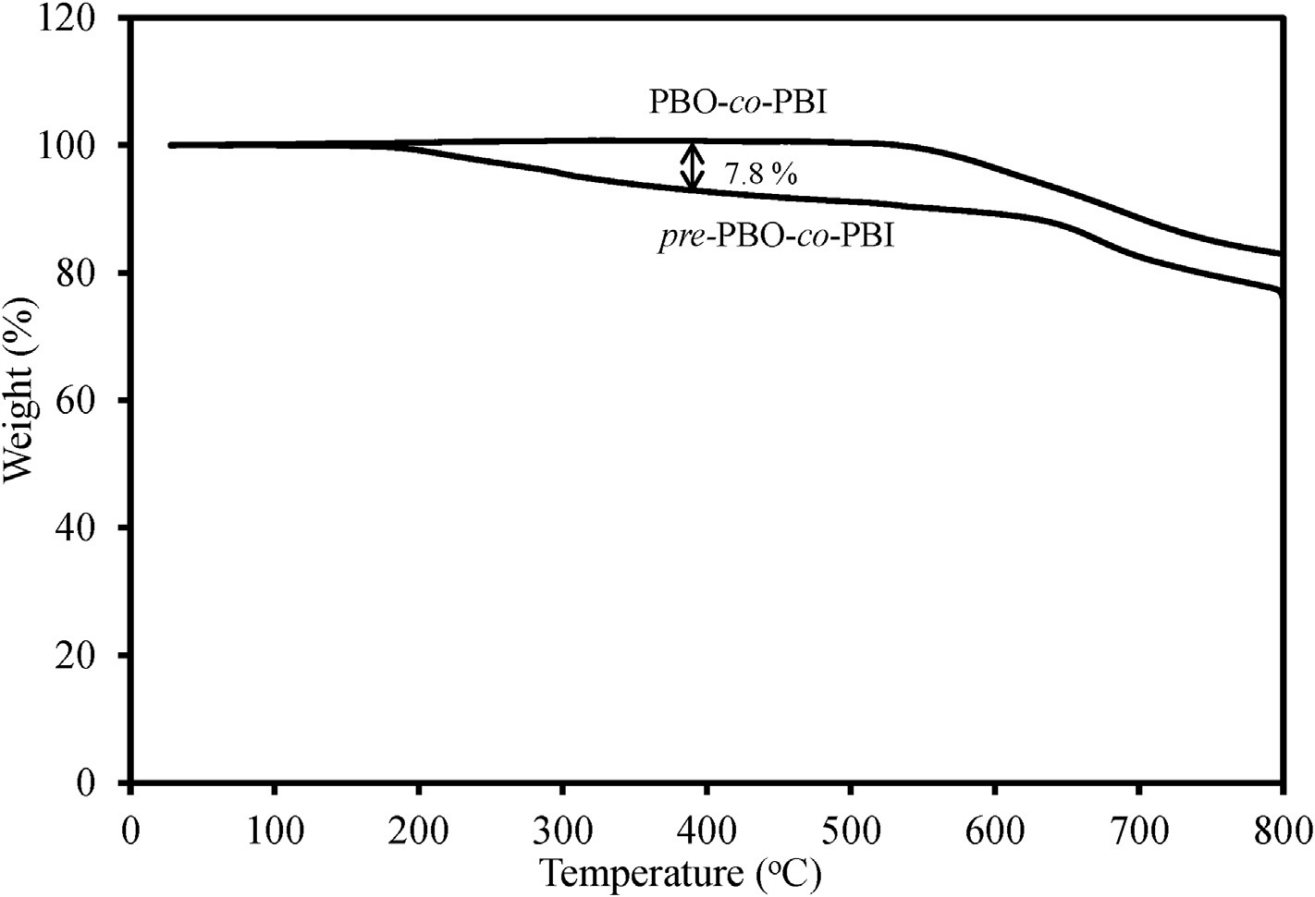
Thermogravimetric curves of PBO-*co*-PBI and its corresponding precursor *pre*-PBO-*co*-PBI.

In Fig. 4 thermogravimetric analysis (TGA) plots were shown, comparing between thermally-cyclized and precursor copolymer for a representative case of all the PBO-*co*-PBIs prepared. Weight-loss was observed between 350 and 400 °C of about 7.8%, certainly due to loss of water molecule as thermal ring closing took place around this temperature range and it was further supported by FT-IR spectra.

**Fig. 5:**
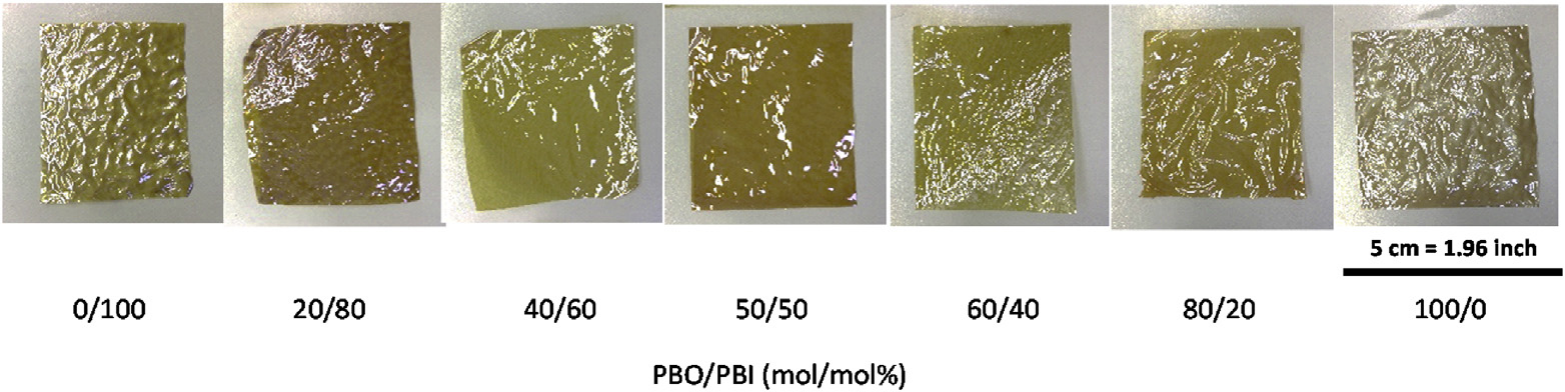
Polymer Film of PBO-*co*-PBI with various compositions (PBO/PBI).

After preparing polymer films using solution casting method, they were washed thoroughly and heated to 400 °C for thermal ring closing, physical texture of the obtained films for all the prepared compositions of PBO-*co*-PBI are shown in Fig. 5. Presence of benz-azole ring and various functional groups in polymer structure, film became yellow in color and non-transparent.

**Fig. 6:**
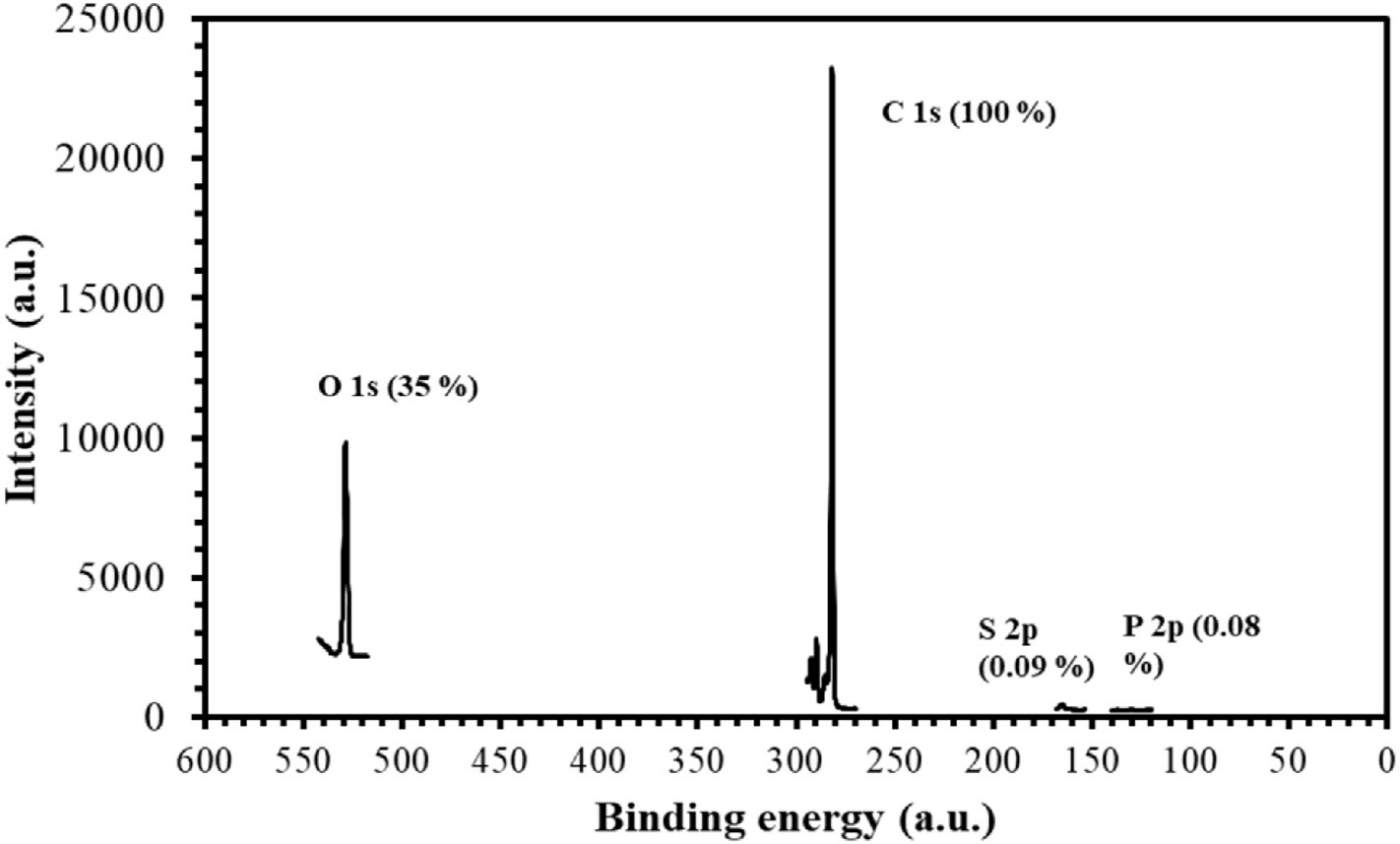
XPS spectra of the polymerized sample to determine elemental analysis.

In Fig. 6 XPS data was shown for a representative case of PBO-*co*-PBI where mainly used for elemental analysis of the amount of remaining solvent. Phosphorus and sulfur content in the polymer films depict presence of solvents such as methanesulfonic acid (MSA)/phosphoric acid which can actually affect the physical properties of the polymer. Atomic percentage of phosphorus (0.08%) and sulfur (0.09%) were observed as compared with carbon and oxygen present in the sample [5].

**Fig. 7:**
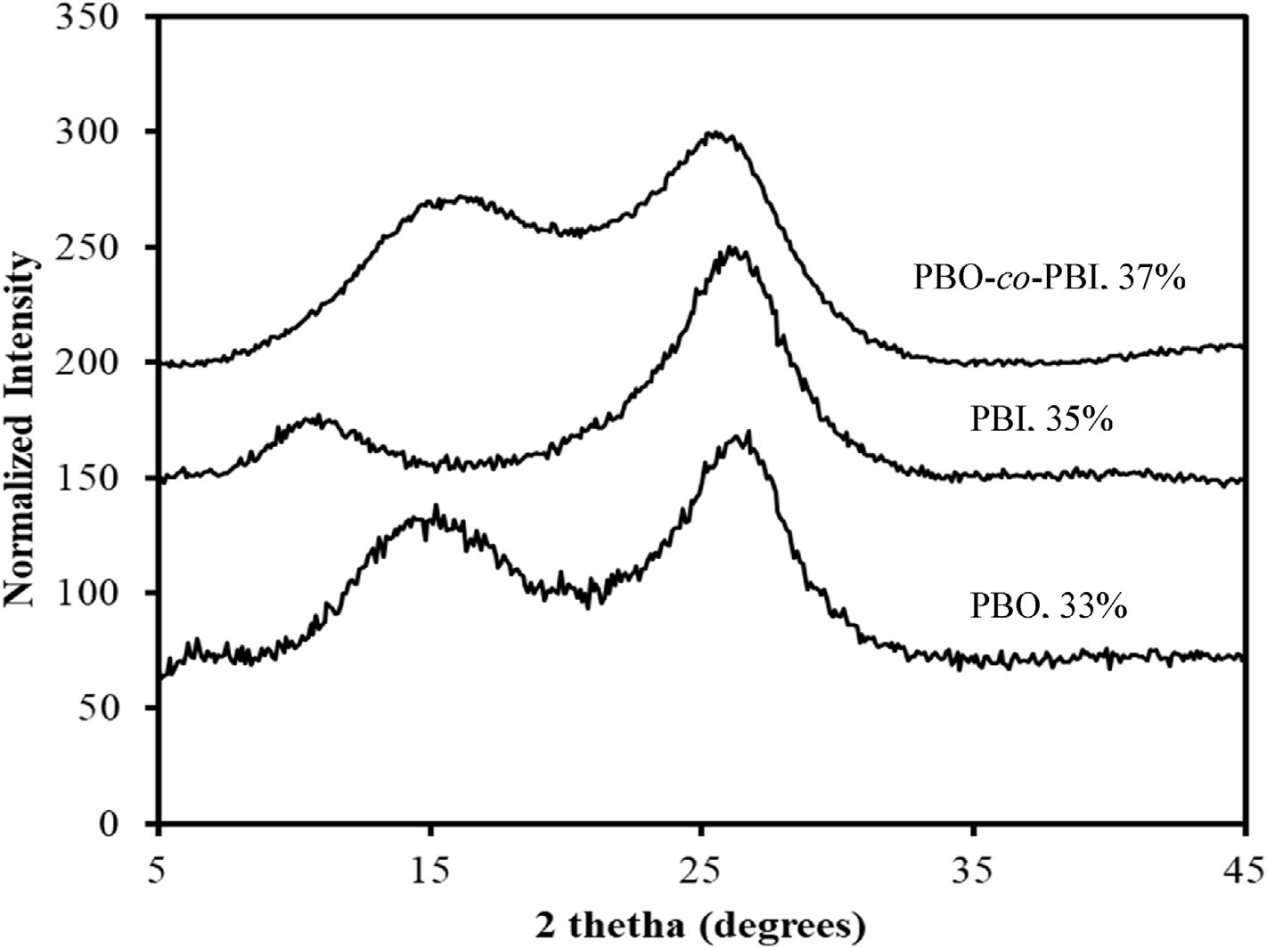
XRD spectra of PBI and PBO and its copolymers PBO-*co*-PBI and PBO/PBI (60/40) compositions.

In Fig. 7 XRD data were shown for the homopolymer (PBO and PBI) and a representative copolymer PBO-*co*-PBI (60/40) along with their degree of crystallinity, which were calculated quantitatively using the method described by Nara and Komiya et al. [3]. The diffraction curves showed two differential peaks both in the case of homopolymer and copolymers which confirms that copolymerization does not affect over crystallinity.

In Table 1, the dielectric data for all the prepared PBO-*co*-PBIs were presented in tabular form for better understanding and elaborated discussion was already done in the main manuscript. All the data were compared with Kapton™, which is a well-known commercial polymer dielectric material [6].

**Table 1:** Dielectric properties for polymers with various PBO/PBI compositions.

Polymers ^a^	Film Thickness (μm)	$\varepsilon^{b}$	$\delta_{v}^{c}\left(\Omega cm\right)$(before drying)	$\delta_{v}^{c}\left(\Omega cm\right)$(after drying)	*D*^d^ (kV/mm)
(PBO/PBI) 0/100	19	3.5	1.8 × 10^8^	2.0 × 10^12^	40.0
20/80	22	3.0	7.9 × 10^9^	7.6 × 10^13^	41.8
40/60	25	2.8	7.8 × 10^8^	3.4 × 10^13^	39.6
50/50	24	2.6	1.9 × 10^9^	6.9 × 10^13^	41.6
60/40	21	2.4	1.3 × 10^10^	2.3 × 10^14^	50.5
80/20	23	2.2	1.9 × 10^10^	1.2 × 10^14^	63.5
100/0	19	1.9	4.3 × 10^10^	1.7 × 10^15^	110.5
Kapton™	25	3.3	5.1 × 10^10^		116.0

(a) Polymer films with various (PBO/PBI) compositions were prepared maintaining film thickness 20 ± 5 μm. (b)Dielectric constant, ε, measured at 1 MHz, (c) Resistivity, ϱv, of the films measured at 1 kV of DC electric voltage for 20 s application, (d) Dielectric strength, D, measured with increasing DC electric voltage up to 6 kV by two terminals method with a ramp-up time of 120s.

## 2 Experimental design, materials and methods

For ^1^H NMR DMSO-*d*_6_ were used as NMR solvent at 23.1 °C with 16 accumulation scans, using proton resonance of residual non-deuterated DMSO as an internal standard (2.55 ppm). PBO-*co*-PBI powder were obtained by stepwise heating for thermal cyclization up to 400 °C and used for FT-IR analysis within the range between 4000 and 400 cm^−1^. TGA was performed at a heating rate of 10 °C min^−1^ under N_2_ atmosphere and for certain cases preheating were done inside TGA instrument to complete thermal cyclization if required. To analyze TGA data, percentage weight loss calculated from the raw data and plotted against temperature. Polymer films were casted following solvent casting method in trifluoroacetic acid (TFA) with 2–3% of MSA. Thickness of the films was maintained in a range between 20 ± 5 μm as dielectric properties of the polymer depend on thickness. The XRD patterns of the samples were analyzed using X-ray diffraction (Rigaku Smartlab) operated at 40 kV, 30 mA with Cu-Kα radiation (1.5418 Å). For X-ray photoelectron spectroscopy (XPS), the polymer film attached on carbon tape and measurements were performed on a Shimadzu Kratos AXIS-ULTRA DLD high performance XPS system. We checked the elemental presence of C 1s and O 1s with respect to the S 2p and P 2p in terms to check the presence of residual solvents if any and thus purity of the sample.
